# Incidence of Respiratory Syncytial Virus Infection Among Patients Aged Less Than 2 Years: A Retrospective Study From an Italian Southern Region in Years 2018–2023

**DOI:** 10.1111/irv.70224

**Published:** 2026-05-14

**Authors:** Giuseppe Di Martino, Pamela Di Giovanni, Federica Vaccaro, Francesca Romana Cascavilla, Carla Di Marzio, Livia Tognaccini, Edoardo Trebbi, Teresa Aita, Ferdinando Romano, Tommaso Staniscia

**Affiliations:** ^1^ Department of Medicine and Ageing Sciences “G. d'Annunzio” University of Chieti‐Pescara Chieti Italy; ^2^ Unit of Epidemiology and Health Statistics Local Health Authority of Pescara Pescara Italy; ^3^ Post‐Graduate School of Hygiene and Preventive Medicine “G. d'Annunzio” University of Chieti‐Pescara Chieti Italy; ^4^ School of Public Health “La Sapienza” University of Rome Rome Italy; ^5^ Local Health Authority of L'Aquila L'Aquila Italy; ^6^ Department of Public Health and Infectious Diseases “La Sapienza” University of Rome Rome Italy

**Keywords:** epidemiology, hospitalization, Italy, preventive medicine, respiratory syncytial virus

## Abstract

**Introduction:**

Respiratory syncytial virus (RSV) is one of the most common viruses infecting nearly all children worldwide within the first 2 years of life, requiring hospitalization for over 80% of cases in infants under the age of 1. The aim of this study was to evaluate the epidemiological impact of RSV infections on hospital admissions among patients aged less than 2 years in the Abruzzo region, Italy. In addition, the analysis on patients aged less than 1 year and the evaluation of direct costs related to RSV admissions were also performed.

**Methods:**

A retrospective observational study was performed in Abruzzo, a Southern Italian region. Data related to hospitalizations were extracted from the hospital discharge record (HDRs) of the Abruzzo region, considering all hospitalizations registered from 2018 to 2023. HDRs from patients younger than 2 years of age and diagnosed with RSV were extracted. Hospitalizations were stratified by calendar year and seasonal cycle. In addition, the analysis of costs generated by each admission was performed.

**Results:**

Totally, 1899 hospitalizations for RSV (98.43 admissions/1000 inhabitants aged under 2 years of age) were recorded in the Abruzzo region during the study period. The median length of stay of all the considered hospitalizations was 5 days (IQR 5–5). The year with the highest number of hospitalizations was 2023. The incident rate among patients under 2 years of age rose from 17.42 to 19.39 in 2023 in A2 over 1000 inhabitants. About costs, the total expenditure for hospitalizations strongly increased from €579,047.29 in 2018 to €1,060,018.94 in 2023.

**Conclusions:**

This study highlights the heavy burden of RSV on hospital admissions among patients aged under 2 years in a region of Southern Italy. These results are in line with previously published research, leading to the necessity of the introduction of effective preventive measures.

## 1 Introduction

Respiratory syncytial virus (RSV) is one of the most common viruses infecting nearly all children worldwide within the first 2 years of life, and it causes most respiratory infections and bronchiolitis requiring hospitalization for over 80% of cases in infants under the age of one [1]. RSV is also a significant pathogen in the elderly population [2].

It is estimated that globally, all individuals are infected by RSV at least once within the first 2 years of life, leading to annually approximately 3 million hospitalizations and 118,000 deaths [3].

RSV is highly contagious, particularly during the colder seasons, with a basic reproductive number (R0) of 3 (±0.6) [4]. RSV circulation depends on geographic location and climate characteristics [5]. In Italy, the virus spreads from November to March with a peak incidence in December–January [6], so RSV infections are typically characterized by marked seasonality, partially according to the flu season [7].

Infected children produce aerosols that might transport RSV through extremely small droplets leading to airways invasion via the nasopharyngeal or conjunctival mucosa. RSV also affects the lower respiratory tract, leading to clinical manifestations such as bronchiolitis and/or pneumonia [4].

The infection typically starts with influenza‐like symptoms, like cough, nasal congestion, rhinorrhea, tachypnea, wheezing, dyspnea, and otitis media. Infants under the age of 2 years have a higher risk of developing inflammations of the smallest lung airways and breathing difficulties [3].

There are also high‐risk groups with a higher susceptibility to severe RSV disease, resulting in hospital admission, increased morbidity and mortality rates. Higher risk groups include preterm infants, small for gestational age infants (SGA), patients with chronic lung disease, bronchopulmonary dysplasia, hemodynamically significant congenital heart disease, immunocompromised conditions, or severe neuromuscular disease. Other known risk factors for hospitalization among patients with RSV are low birth weight, male gender, presence of an older sibling, exposure to smoking, young maternal age, and living in a suburban context [8].

Furthermore, infections contracted during the first year of life, even without hospitalization, can increase the long‐term risk of developing a heightened susceptibility to respiratory diseases, such as asthma [9]. A recent study showed that children hospitalized in the first 2 years of age had a three‐fold higher risk of asthma hospitalization and greater use of anti‐asthmatic drugs [10], leading to a substantial healthcare burden and economic costs associated with RSV disease.

Medical care for RSV disease is based on unspecific therapy, including mainly supportive measures, such as oxygen delivery, hydration, and antipyretic administration [11]. Therefore, based on the literature, over the past few years, the development of treatments has focused on preventive strategies such as monoclonal antibodies (mAbs) and adult and maternal vaccines [12].

A recent systematic review analyzed different papers about RSV‐associated hospitalizations in children under 6 years of age in Italy [13]. It is evident that most hospitalizations involved patients < 1 year of age. In particular, for this age group, the hospitalization rate resulted in a range of 71.5% to 88.8% [14, 15, 16], reaching a peak during January and February [15, 17].

During last years, innovative prophylactic strategies against RSV infections, such as a new monoclonal antibody (Nirsevimab–Beyfortus) and a vaccine for neonatal passive immunization (Abrysvo) administered via maternal vaccination during pregnancy, were introduced, strongly impacting RSV epidemiology [18].

The aim of this study was to evaluate the epidemiological impact of RSV infections on hospital admissions among patients aged less than 2 years in the Abruzzo region, Italy. In addition, the analysis on patients aged less than 1 year and the evaluation of direct costs related to RSV admissions were also performed. It is important to highlight that recent data from the Abruzzo region on this topic were not available and pharmacological preventive measures were still not introduced.

## 2 Materials and Methods

A retrospective observational study was performed in Abruzzo, a Southern Italian region counting about 1.3 million inhabitants [19]. Data related to hospitalizations were extracted from the hospital discharge record (HDRs) of the Abruzzo region, considering all hospitalizations registered from January 1, 2018, to December 31, 2023.

HDRs contain various data regarding information on patient demographic characteristics, such as age, gender, city of residence, and marital status. In addition, it was included a diagnosis‐related group (DRG) code used to classify the admission, with up to six diagnoses (one principal diagnosis and up to five comorbidities) and up to six possible procedures performed during the hospitalization.

Diagnoses and procedures were coded according to the International Classification of Disease, 9th Revision, Clinical Modification (ICD‐9‐CM) system, the National Center for Health Statistics (NCHS), and the Centers for Medicare and Medicaid Services External, Atlanta, GA, USA.

In the study period, HDRs from patients younger than 2 years of age and diagnosed with RSV acute bronchiolitis (ICD‐9‐CM code 466.11), RSV pneumonia (ICD‐9‐CM code 480.1), and RSV positivity (ICD‐9‐CM code 079.6) (A1 analysis group) were selected.

Additionally, a second analysis (A2), involving the ICD‐9‐CM code 466.19 (acute bronchiolitis by unspecified organism), was performed. Indeed, a microbiological confirmation diagnosis is sometimes not obtained during hospitalization, or incorrect coding occurs during the compilation of the HDRs. It is important to highlight that approximately 60% of associated diagnoses with this code are attributable to RSV, according to literature [20].

Hospitalization data were stratified by calendar year (from January to December of each year) and seasonal cycles (from June of a year to May of the following one). The analysis was focused on the hospitalization of patients under 1 year of age, which represents the age class of higher risk of admission. In addition, neonatal intensive care unit hospitalizations were investigated. The length of stay was expressed as median and interquartile range (IQR).

Regarding the cost analysis of hospitalizations, the tariff, expressed in Euro (€), corresponding to the DRG generated by each individual hospitalization, was considered.

Data analysis was performed using Stata v20 software (Stata Corp., TX, USA).

### 2.1 Ethical Statement

The study was conducted in conformity with the regulations on data management of the Regional Health Authority of Abruzzo and with the Italian Law on privacy (Art. 20–21 DL 196/2003), published in the Official Journal, n. 190, on August 14, 2004. The data were encrypted prior to the analysis at the regional statistical office, when each patient was assigned a unique identifier. The identifiers eliminated the possibility of tracing the patients' identities. According to Italian legislation, the use of administrative data does not require any written informed consent from patients.

## 3 Results

**FIGURE 1:**
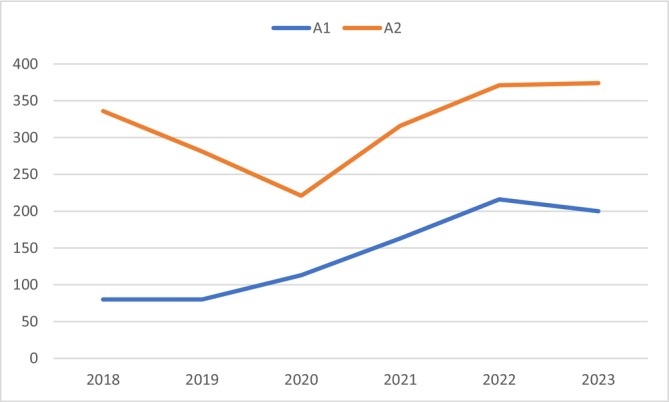
Hospitalization trends for RSV and bronchiolitis between 2018 and 2023.

Between 2018 and 2023, a total of 1899 hospitalizations for RSV (98.43 admissions/1000 inhabitants aged under 2 years of age) and bronchiolitis due to unspecified microorganism (A2) were recorded in the Abruzzo region, including 852 (44.9%—44.16 admissions/1000) with specific codes for RSV (A1). The median length of stay of all the considered hospitalizations was 5 days (IQR 5–5). All patients were discharged at home and no patients died during the study period. No patients reported comorbidities at the time of the admission, so all admitted subjects were healthy prior to the hospitalization. The year with the highest number of hospitalizations was 2023, with 374 total admissions, including 200 (53.5%) with specific RSV codes (Figure 1). The incident rate among patients under 2 years of age rose from 4.15 in 2018 to 10.37 in 2023 for A1 and from 17.42 in 2018 to 19.39 over 1000 inhabitants in 2023 in A2.

**FIGURE 2:**
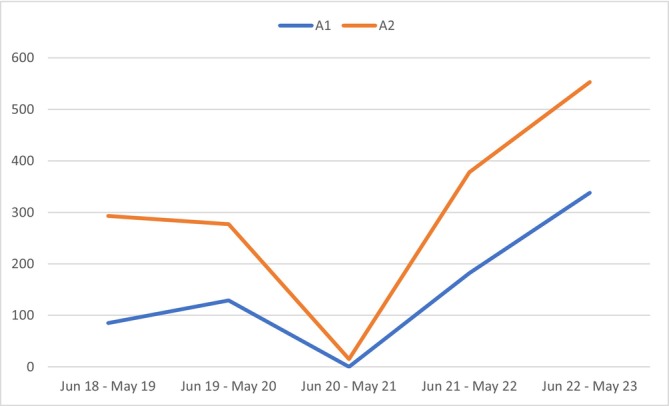
Seasonality hospitalization trends for RSV and bronchiolitis between 2018 and 2023.

Considering the seasonality of RSV, the period between June 2022 and May 2023 showed the highest number of hospital admissions, with 553 admissions in group A2, including 338 (61.1%) with RSV diagnosis (Figure 2).

Focusing on patients under 1 year of age, a total of 1708 hospitalizations were recorded in A2 and 728 in A1 (42.6%). Table 1 shows the hospitalization distribution based on the month of admission.

**TABLE 1:** Hospitalization distribution in patients < 1 year of age by admission month.

Month	A1		A2	
	*n*	%	*n*	%
January	162	22.25	351	20.55
February	119	16.35	304	17.80
March	50	6.87	155	9.07
April	11	1.51	78	4.57
May	5	0.69	43	2.52
June	4	0.55	18	1.05
July	1	0.14	14	0.82
August	8	1.10	18	1.05
September	112	15.38	18	1.05
October	256	35.16	56	3.28
November	162	22.25	216	12.65
December	119	16.35	437	25.59

Abbreviations: A1 = admissions for RSV specific infection; A2 = admissions for acute bronchiolitis by unspecified organism.

**FIGURE 3:**
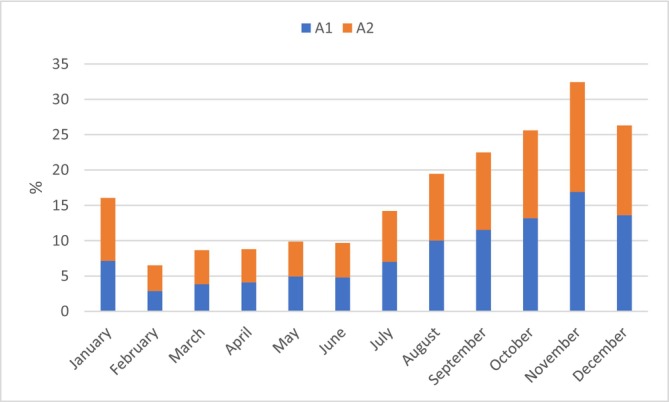
Hospitalization distribution in patients < 1 year of age by birth month.

Considering the birth month in relation to hospital admission, Table 2 showed that patients born between August and January are at a higher risk of admission, as shown also in Figure 3.

**TABLE 2:** Hospitalization distribution in patients < 1 year of age by birth month.

Month	A1		A2	
	*n*	%	*n*	%
January	52	7.14	152	8.90
February	21	2.88	62	3.63
March	28	3.85	82	4.80
April	30	4.12	80	4.68
May	36	4.95	84	4.92
June	35	4.81	83	4.86
July	51	7.01	123	7.20
August	73	10.03	161	9.43
September	84	11.54	187	10.95
October	96	13.19	212	12.41
November	123	16.90	265	15.52
December	99	13.60	217	12.70

Abbreviations: A1 = admissions for RSV specific infection; A2 = admissions for acute bronchiolitis by unspecified organism.

Regarding intensive care unit (ICU) admissions in patients under 1 year of age, 50 admissions (61.7%) were recorded in A2, while 31 admissions (38.3%) were referred to A1. The median length of stay was 10 days (IQR 7–20). The age groups most frequently requiring ICU admission were those up to the third month of life, as shown in Table 3.

**TABLE 3:** ICU admission distribution by month of life in patients under 1 year of age.

Age in month	A1		A2	
	*n*	%	*n*	%
< 1	7	20.00	12	16.67
1	21	14.00	29	9.09
2	3	2.34	5	1.65
3	0	0.00	2	0.93
4	0	0.00	1	0.58
5	0	0.00	1	0.71
6	0	0.00	0	0.00
7	0	0.00	0	0.00
8	0	0.00	0	0.00
9	0	0.00	0	0.00
10	0	0.00	0	0.00
11	0	0.00	0	0.00
Total	31	100.00	50	100.00

About costs, as reported in Table 4, the total expenditure for hospitalizations strongly increased from €81,805.76 in 2018 to €592,276.38 in 2023 considering A1. In A2, the expenditure increased from €579,047.29 in 2018 to €1,060,018.94 in 2023.

**TABLE 4:** Costs of RSV‐related hospitalizations by year.

	A1					A2				
	Mean	Median	Min	Max	Total	Mean	Median	Min	Max	Total
2018	802.37	1501.86	0	3712.58	81.805,76	1402.64	1501.86	0	3712.58	579,047.29
2019	842.02	1501.86	0	8160.32	81.954,93	1376.53	1501.86	0	30,016.08	516,488.95
2020	1834.81	1501.86	0	30,016.08	217.100,98	1983.88	1501.86	0	30,016.08	490,231.87
2021	1079.62	1501.86	0	30,016.08	260.269,89	1490.47	1501.86	0	30,016.08	585,520.60
2022	1533.92	1501.86	0	30,016.08	455.453,35	1661.24	1501.86	0	30,016.08	778,968.82
2023	2691.5	1501.86	0	30,016.08	592.276,38	2454.62	1501.86	0	30,016.08	1,060,018.94
Total					1,688,861.29					4,010,276.47

## 4 Discussion

This paper presented trends in RSV‐related hospitalizations among patients under 2 years of age in the Abruzzo region between 2018 and 2023 [13, 16]. As reported by previous studies, also in recent years, RSV represents a heavy burden among patients during the first 24 months of life, also among healthy infants. In addition, this study confirmed the low mortality rate previously reported in the literature [13, 14, 16].

The reported hospitalization rate was similar to a previous study performed in Europe. In particular, a study from Sweden reported an incident rate of 17.4/1000 among children under 1 year of age [21]. A study from Spain reported a higher rate, counting 24.13/1000 inhabitants [22]. The pandemic season from June 2020 to May 2021 reported the lowest incidence (15 admissions in A2 analysis). This period included the first pandemic year that was characterized by heavy use of facial masks, social distancing, and lockdown that limited viruses circulation [23]. Several studies confirmed the decrease in RSV‐related infections and admissions, ranging from 80.8% to 99.6% [23, 24]. However, it is well known that RSV represents the most frequent cause of hospital admission among patients under 2 years of age [25]. The strong increase in hospitalization after the pandemic could be linked to immunity debt and the role played by non‐pharmacological interventions used during the pandemic in reducing viral infections. The increase during the last years after the pandemic in the RSV‐specific admissions compared to unspecified infections could be explained by a more accurate diagnosis.

This paper showed that children aged under 1 year of age represent the higher proportion of hospitalized patients and, in particular, children born from August to January were at higher risk of admission. This was in line with previous literature [21, 26].

Evaluating admissions in ICU, all patients were younger than 3 months, confirming that younger children are at higher risk of developing the most severe disease [18]. However, during the last decades, the management of bronchiolitis was changed thanks to the increased use of non‐invasive respiratory therapy [27]. Despite that, younger children, preterm and patients with comorbidities remain at high risk of ICU admission.

About costs, despite the low number of considered admissions, the cost of hospitalizations was high, reaching over €2500 per patient during year 2023 and over 1.5 million during the entire study period. Poor studies reported the cost of hospitalizations, and this is one of the first studies that highlight the economic burden of RSV‐related hospitalizations. In parallel to the increase in hospital admission, costs also increased during the study period. To date, only one paper from Italy reported direct costs [28], showing higher mean expenditure compared to the present study, as also confirmed by a recent systematic review [8]. However, it is important to highlight that the simple evaluation of the DRG tariff underestimates the real cost of each hospitalization. The tariff cannot consider several aspects of RSV management (specialist evaluation, general practitioner, and emergency room visits), and it cannot consider the indirect costs of RSV infection. In order to evaluate indirect costs related to RSV, it is mandatory to estimate also the mean ambulatory visit cost, the out‐of‐pocket drug cost, and the long‐term follow‐up impact of infected patients. In particular, children with RSV infection during the first 2 years of age have a higher risk to develop asthma [29] within a 5 years period, also in preterm and late‐preterm patients [30]. This condition leads to several ambulatory accesses, drug therapy, and hospital admissions, increasing the costs of a prior RSV infection. Also, long‐term indirect costs are related to the asthma exacerbations during the next years after the primary infection.

The introduction of a mass preventive strategy, such as a long‐acting monoclonal antibody (Nirsevimab), showed a substantial reduction in infant RSV‐related hospitalizations [18]. A recent narrative review [31] summarized recent real‐world evidence on universal prophylaxis with Nirsevimab among infants, reporting significant decreases in hospitalization in several countries [32, 33, 34].

The first Italian region introducing universal preventive strategy was Valle d'Aosta, confirming the efficacy of this prophylactic measure [35]. However, one more possible strategy against RSV infection is maternal immunization during pregnancy with bivalent prefusion F vaccine (RSVpreF, Abrysvo). This alternative strategy was effective towards severe RSV‐related disease [36]. Actually, in Italy, poor regions introduced the RSVpreF in the vaccination schedule. However, several countries introduced this vaccination in their schedule, and some countries included it in their relative guidelines [37]. However, maternal RSV vaccination at 32–36 weeks' gestation is recommended during only September–January in most countries and US states.

## 5 Strengths and Limitations

This study presents several points of strength. First, it was the first evaluation of RSV‐related hospitalizations in the Abruzzo region. The study evaluated a large study period (2018–2023), and it is one of the first studies including the full 2023 year. In addition, this is one of the first studies reporting the direct admission costs of RSV‐related hospitalizations in the Italian setting. However, this study presents some limitations. First, the HDRs are not used for epidemiological purposes but rather for admission‐related remuneration. The evaluation of causes of admission could be underestimated due to miscoding. Second, HDRs lack important patient clinical data, such as drug therapies, blood parameters, and the clinical severity of each illness. The absence of this information influences the depth of our analysis. Finally, the use of HDRs focused only on hospitalized patients, lacking information on out‐of‐hospital RSV cases. This point led to an underestimation of the burden of disease.

## 6 Conclusions

This study highlights the heavy burden of RSV on hospital admissions among patients aged under 2 years in a region of Southern Italy. These results are in line with previously published research, leading to the necessity of the introduction of effective preventive measures.

## Author Contributions

**Giuseppe Di Martino:** conceptualization, methodology, formal analysis, writing – original draft preparation. **Pamela Di Giovanni:** conceptualization, validation, writing – original draft preparation. **Federica Vaccaro:** methodology, software, formal analysis, writing – review and editing. **Francesca Romana Cascavilla:** software, validation, formal analysis, resources, writing – original draft preparation. **Carla Di Marzio:** software, validation, formal analysis, resources, writing – original draft preparation. **Livia Tognaccini:** validation, investigation, data curation, writing – review and editing. **Edoardo Trebbi:** validation, investigation, data curation, writing – review and editing. **Teresa Aita:** software, writing – review and editing, visualization. **Ferdinando Romano:** data curation, writing – review and editing, visualization, supervision, project administration. **Tommaso Staniscia:** conceptualization, writing – review and editing, visualization, supervision, project administration.

## Funding

The authors have nothing to report.

## Ethics Statement

The study was conducted in conformity with the regulations on data management of the Regional Health Authority of Abruzzo and with the Italian Law on privacy (Art. 20–21 DL 196/2003), published in the Official Journal, n. 190, on 14 August 2004. The data were encrypted prior to the analysis at the regional statistical office, when each patient was assigned a unique identifier. The identifiers eliminated the possibility of tracing the patients' identities.

## Consent

According to Italian legislation, the use of administrative data does not require any written informed consent from patients.

## Conflicts of Interest

The authors declare no conflicts of interest.

## Data Availability

Data are available at the Health Department of Abruzzo Region after reasonable request only for scientific purpose.
